# A Data Science Approach to Estimating the Frequency of Driving Cessation Associated Suicide in the US: Evidence From the National Violent Death Reporting System

**DOI:** 10.3389/fpubh.2021.689967

**Published:** 2021-08-16

**Authors:** Tomohiro M. Ko, Viktoryia A. Kalesnikava, David Jurgens, Briana Mezuk

**Affiliations:** ^1^Rutgers—Robert Wood Johnson Medical School, Piscataway, NJ, United States; ^2^Department of Epidemiology, University of Michigan, Ann Arbor, MI, United States; ^3^School of Information, University of Michigan, Ann Arbor, MI, United States; ^4^Institute for Social Research, University of Michigan, Ann Arbor, MI, United States

**Keywords:** driving cessation, suicide, aging, machine learning, natural language processing

## Abstract

Driving cessation is a common transition experienced by aging adults that confers both a symbolic and literal loss of independence due to the central role of automobiles for mobility in the US. Prior research has shown that driving cessation has negative implications for mental health, social participation, and access to healthcare. Given these sequelae of driving cessation and prior work showing that late-life transitions related to independence (e.g., transitioning into residential care) are associated with suicide, we sought to estimate the frequency of driving cessation associated suicide. Data include suicide (*n* = 59,080) and undetermined (*n* = 6,862) deaths aged ≥55 from the National Violent Death Reporting System (NVDRS, 2003–2017). Each case in the NVDRS has both quantitative data (e.g., demographic characteristics) and qualitative text narratives, derived from coroner/medical examiner reports, which describe the most salient circumstances and features of each death. To identify cases associated with driving cessation, we employed a supervised random forest algorithm to develop a Natural Language Processing (NLP) classifier. Identified driving cessation associated cases were then categorized and characterized using descriptive statistics and qualitative content analysis. From 2003 to 2017, there were an estimated 305 cases of suicide/undetermined deaths associated with driving cessation in the NVDRS, representing 0.04% of all cases. Cases associated with driving cessation were older, more likely to be male, more likely to have a physical health problem, more likely to have experienced a recent crisis, and more likely to have lived in a rural county than other decedents. Qualitative analysis identified functional impairment, alcohol-related driving limitations, loss of employment, and recent car accidents as common themes among cases associated with driving cessation. This analysis illustrates the utility of NLP in identifying novel correlates of suicide in later life. Although driving cessation associated suicide is a rare outcome, further research is warranted on understanding the conditions under which driving cessation is associated with suicidal behavior, and how to support the well-being of aging adults during these types of major life transitions.

## Introduction

Suicide is the 10th leading cause of mortality in the United States, and in 2019, 47,511 individuals died by suicide (1). Aging adults have among the highest rate of suicide, particularly among non-Hispanic white (NHW) men (2). Over the past decade, NHW men aged 55 and older have an age adjusted suicide rate of 32.96/100,000 as compared to 23.04/100,000 for NHW men aged 15–25 (3).

Aging is a process marked by substantial transitions across social, health, and psychological domains. For many, it is a process that often entails a loss of independence. Contributors to this loss of independence include the exacerbation of chronic health conditions, impairments in vision, hearing or cognition, impairments in mobility, and potentially needing support managing medical conditions or activities of daily living, whether in home or in residential care (4–6). Whether and how this loss of independence relates to suicide risk in later life is not well-understood. However, a growing body of research suggests that transitions related to loss of independence are correlated with suicide risk. For example, a recent study estimated that 2.2% of suicide deaths among adults aged ≥55 were related to long-term care in some manner, with the largest proportion of cases related to transitions into or out of residential long-term care (7). This suggests that late-life loss of independence, in this case a loss of independent living, may be an important factor in suicide among aging adults.

As a result of accumulating medical and functional impairments, many aging adults experience a decline of independence related to mobility in the form of driving cessation. Driving is by far the predominant and preferred method of transportation in the United States (8). However, driving cessation is common in later life; over 600,000 adults over the age of 75 stop driving each year (9). In 2018, ~1 in 5 adults aged 65 and older in the U.S. did not have a valid driver's license (10). Driving cessation is not just something that occurs at the end of life; persons typically live close to a decade longer after they stop driving (9, 11).

Reduction in or cessation of driving in later life can occur for a variety of reasons including functional limitations and voluntary self-regulation (e.g., not driving at night or in bad weather). Changes in driving also occur as a result of coercion from others, including the urging of family members and healthcare staff (12–14). Cessation can also occur involuntarily through being unable to renew licensure. A 1995 California Department of Motor Vehicles (DMV) report noted that among 3,669 randomly selected license renewal applicants, 5% of 40–51 year olds, 7% of 52–69 year olds, and 25% of 70+ year olds failed the CA DMV standard Snellen Vision Test (15).

Driving cessation, whether voluntary or involuntary, is associated with decreased quality of life, depression, isolation, and reduced network of friends and access to health care (16–18). These experiences were expressed clearly in a qualitative study of older former-drivers, which revealed themes related to resentment toward increased dependence on others for transportation, inconvenience, loss of control over personal schedule, and activity reduction (12). The ability to drive is also an important social role in many aging adults, and cessation entails a “reduction of status and freedom, independence and opportunity” (19–21).

Given the negative effects of driving cessation on sense of autonomy and independence, there is reason to believe that aging adults may experience an increased risk of suicide as a result of driving cessation. The Interpersonal Theory of Suicide posits that perceived burdensomeness, thwarted belongingness, and acquired capability for suicide are jointly causative of lethal suicidal behavior in later life (22, 23). Functional impairments elevate the risk of feeling that “My death is worth more than my life to others,” or perceived burdensomeness, while social isolation elevates the risk of thwarted belongingness—an unmet need to belong; habituation to pain from chronic illness increases the acquired capability for suicide (24). Aging adults who have stopped driving report feeling like a burden on friends and family who transport them, and may have functional impairments and chronic illnesses that precipitated driving cessation (25–29). Thus, there may be an elevated risk of suicide associated with driving cessation among aging adults.

Although the relationship between driving cessation and depression is well-documented, to our knowledge, the frequency of suicide related to driving cessation among aging adults has never been estimated (30–32). Therefore, the present analysis aims to (1) Estimate the frequency of driving cessation associated suicide among aging adults, (2) describe the salient circumstances and features of decedents of suicide associated with driving cessation using quantitative and qualitative approaches, and (3) discuss the utility of a supervised random forest algorithm in identifying driving cessation associated suicides.

## Methods

### Data Source

This analysis uses Restricted Access Data (RAD) from the Centers for Disease Control and Prevention (CDC) National Violent Death Report System (NVDRS), a state-based registry of violent deaths (homicides, firearm accidents, undetermined deaths, and suicides) started in 2003. Initially, six states reported deaths to the NVDRS; as of 2017, 37 states reported deaths to the NVDRS (33). The present analysis used a subset of deaths from suicides (*n* = 59,080) and undetermined (*n* = 6,862) deaths among adults age 55 and older that occurred from 2003 to 2017 (total analytic sample *N* = 65,942).

This RAD project was approved by the CDC and was deemed exempt from human subjects regulation by the University of Michigan Institutional Review Board as all participants in the NVDRS are deceased.

#### Quantitative Variables

A variety of quantitative variables related to the decedent's demographic characteristics as well as circumstances of death were incorporated in the present analysis from the NVDRS. These variables were coded by NVDRS staff from original source documents (e.g., death certificates, medical examiner reports, law enforcement investigations, suicide notes) using CDC-developed data entry software. Variables include sex, age, race, years of education, marital status, history of a physical health problem, history of a mental health problem, the presence of a recent crisis preceding the suicide, history of suicidal ideation, and whether suicidal thoughts were disclosed to another individual.

In order to proxy the relative reliance on automobiles for transportation, we also merged the NVDRS registry data with the 2013 county-level Rural Urban Continuum Code (RUCC), developed by the US Department of Agriculture using county-level identifiers in both data sources. RUCCs are primarily based on population and metropolitan areas; they range from a maximum code of 1- “Counties in metro areas of 1 million population or more” to a minimum code of 9- “Completely rural or <2,500 urban population, not adjacent to a metro area.” For example, Baltimore City (part of the Baltimore-Washington metro area) has a RUCC code of “1,” whereas Jefferson County, MT–with a population of <12,000–has a RUCC code of “9.” Further documentation on their development is described elsewhere (34).

#### Medical Examiner/Coroner Narrative Reports

The RAD NVDRS data include text narratives for each case in the registry. These narratives are written by trained NVDRS state coders using original source documents (e.g., medical examiner/coroner reports, law enforcement investigations). The dataset provided by the NVDRS was structured such that the narratives were included as a free text variable (i.e., occupying one column on the spreadsheet) alongside the aforementioned quantitative variables. Further details on the process for data abstraction in these narratives is described elsewhere (7, 35).

The narratives abstracted from the medical examiner/coroner reports are more complete than those written from other sources (e.g., not all deaths, particularly suicides, are investigated by law enforcement), and therefore these are the narratives used to qualitatively describe cases related to driving cessation. Per CDC reporting guidelines, all excerpts and samples of narrative text in this manuscript are paraphrased amalgamations of multiple narratives to protect privacy.

### Analysis

The NVDRS has an enormous breadth of data on the circumstances surrounding each death in the registry; however, it lacks variables indicating driving status (current vs. former vs. never) and mobility transitions, and neither loss of license nor vehicle qualify as a “recent crisis” in the NVDRS coding schema. However, the RAD narrative texts often describe such events and characteristics, and they can potentially be leveraged to identify cases related to cessation. This is similar to challenges related to identifying suicide deaths relate to residential transitions (i.e., moving into or out of long-term care) that were addressed in our prior work (7). Building on this experience, in this paper we employed a supervised random forest algorithm to develop a Natural Language Processing (NLP) classifier that estimates the frequency of driving cessation associated suicide from the coroner/medical examiner narratives.

We employed a stepwise, iterative procedure for identifying deaths associated with driving cessation from the NVDRS narrative texts, summarized briefly here and described in detail below:

- Create a set of labeled “training” data (e.g., text narratives where the case was determined to be associated with driving cessation vs. cases not associated with driving cessation) through a manual process of reading a sample of the narratives identified by a keyword search;
- Pre-process all narrative texts to prepare them for NLP analysis;
- Train the initial random forest classifier with the labeled “training” data on the remaining unlabeled “testing” data;
- Review and label the results from the initial model to create a new, larger labeled dataset to improve the classification accuracy of the random forest classifier;
- Train a second text classification model on the expanded labeled dataset to improve its performance at classifying cases associated with driving cessation vs. those not associated with driving cessation;
- Use the results from the second model to identify cases of driving cessation associated suicides vs. those not associated with driving cessation;
- Conduct a manual search of the text of cases labeled as not related to driving cessation to identify those that may have been incorrectly missed by the classifier.

**Figure 1:**
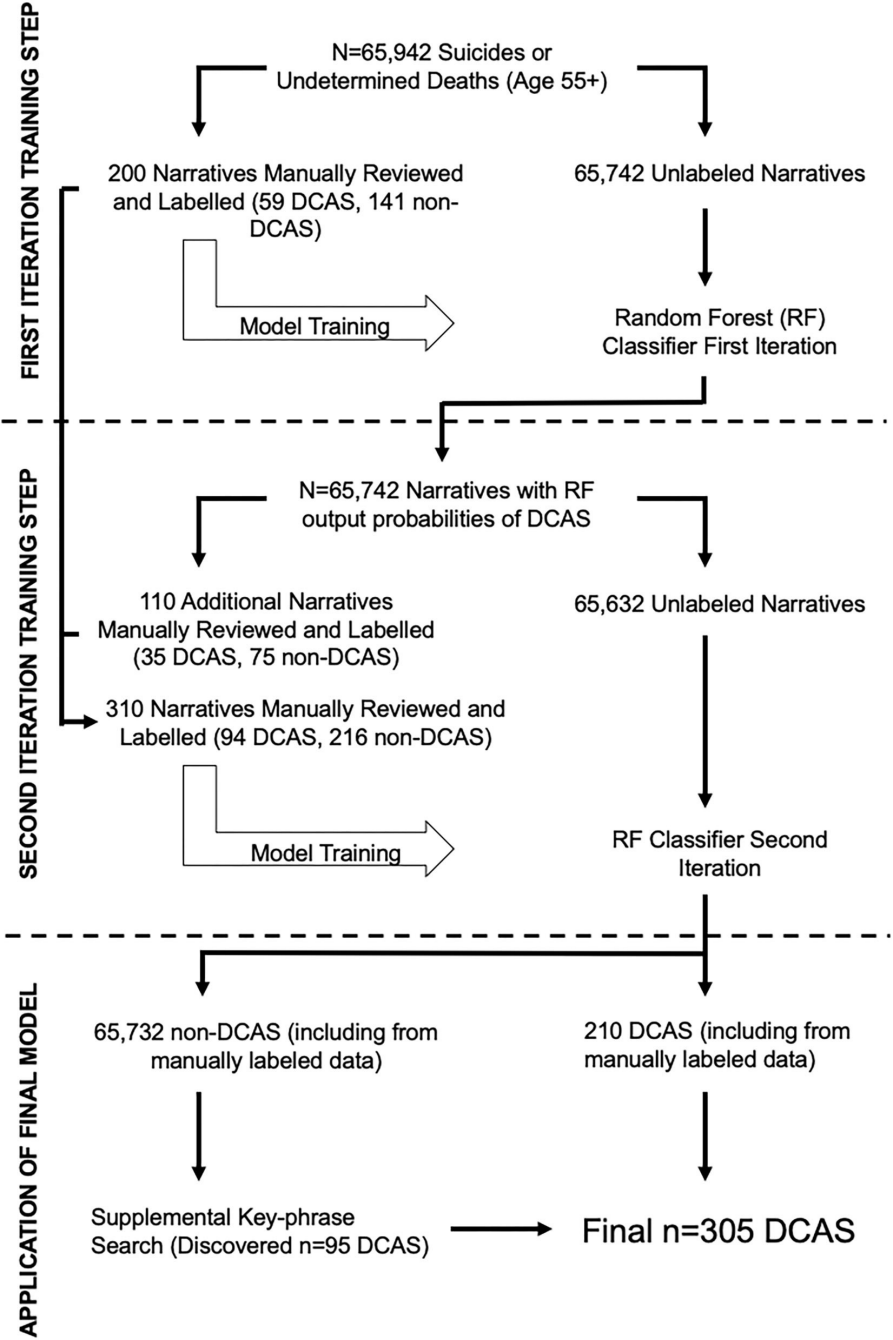
The stepwise, iterative procedure used to develop the Natural Language Processing (NLP) classifier, which employs a supervised random forest algorithm, is illustrated. A supplemental key-phrase search was conducted to identify cases that may have been incorrectly missed by the classifier. DCAS denotes driving cessation associated suicide.

See Figure 1 for a flowchart summarizing this stepwise procedure. All analyses were conducted in R v 4.0.3; caret, tm, and RWeka were used for machine learning and NLP analyses.

#### Creation of the Initial Labeled Training Dataset

Based on our prior experience with this dataset, we expected that narratives that described driving cessation as a salient circumstance would be rare in the NVDRS. Therefore, we began with a keyword search of all medical examiner/coroner narrative texts to develop a manually-labeled dataset with a sufficient balance of cases related to driving cessation vs. cases not related to driving cessation. This approach to creating the training data contrasts with simply labeling a randomly selected subset of the data (an efficient way to minimize bias in a model), which can be appropriate when cases are expected to be frequent.

All narratives were searched for the presence of four keywords likely to capture driving cessation associated suicide: “drive,” “license,” “vision,” and “DMV.” Narratives were separated into bins depending on the extent of keyword matching. Sixty (*n* = 60) narratives were randomly selected and manually reviewed from the largest bins (i.e., there were *n* = 213 texts that matched only “drive”; *n* = 123 texts that matched only “vision”; and *n* = 313 texts that matched only “license”). The remaining bins with at least one keyword match had an n ≤ 10, and thus were all manually reviewed. Finally, to better reflect the balance of driving cessation associated suicide in the data, 34 narratives were randomly selected and reviewed from the “likely unrelated to driving cessation case” bin (i.e., from texts that did not match any of the keywords). In total, the first labeled training dataset consisted of 200 narratives: 59 cases related to driving cessation and 141 cases unrelated to driving cessation.

All manual reviews of narratives were conducted by TK, but the annotation process of reviewing them was discussed by the entire study team. In order to be considered “driving cessation-associated,” explicit mention of the driving cessation was necessary, i.e., inferred or implicit driving cessation was not sufficient. Any explicit mention of an inability to drive, avoidance of driving, pressure to stop driving, or declining ability to drive was considered driving cessation. Further, driving cessation could be currently instated, transient, or expected, regardless of the reason. As our analysis is not causal, we did not require the suicide to be explicitly attributed to driving cessation. We used these criteria in order to produce a conservative estimate and model of driving cessation, as extensive subjective inference could artificially inflate the frequency of the outcome. Suicide deaths wherein the narrative met the aforementioned criteria for driving cessation are referred through the rest of this manuscript as DCAS (Driving Cessation Associated Suicide).

For example, the following decedent narrative would be indicated as DCAS: “76 year old white male was found dead in his living room from a gunshot wound to the head. The victim was depressed over medical issues, including several strokes, and was recently told he would no longer be allowed to drive. The victim told family members that he would kill himself if he could not drive. The victim was dependent on family for daily matters and had a history of depression and hypertension.” On the other hand, the following decedent narrative would be indicated as non-DCAS: “75 year old white male was found deceased due to an intraoral gunshot wound in the driver's seat of his vehicle. The victim's family noted that he had been depressed since his wife passed away 2 years ago. The victim had also been suffering from chronic pain over the past several years.” As mentioned previously, these narratives are paraphrased, de-identified amalgamations of multiple narratives to protect privacy.

#### Narrative Text Pre-processing and Tokenization

The narrative texts were prepared through a series of standard NLP pre-processing (36). This included (a) converting all text to lower-case; (b) removing all text formatting, punctuation, and stop words (i.e., uninformative words (e.g., a, and, are, as, at,) that do not confer any advantages in distinguishing pieces of text); and (c) stemming all words (i.e., decomposed to the root: “walking,” “walked,” “walks” would all be stemmed to “walk”) to reduce unmeaningful word variation.

Next, this pre-processed text was broken into analyzable units, a process called *tokenization*. The size of tokens is determined by the user, and can range from single letters to full paragraphs. The present analysis uses unigrams and bigrams, which are single words and successive two-word phrases. From the phrase “white male drove,” the derived unigrams would be “white” “male” and “drove,” whereas the bigrams would be “white male” and “male drove” (37). The data was then re-structured into a document term matrix, where each row is a document (a given case narrative), and columns represent tokens (unigrams and bigrams), with fields populated by the frequencies of the token in a given document. Then, the frequencies of the tokens were weighted via Term-Frequency Inverse Document Frequency (TF-IDF) (38). TF-IDF captures the importance of a token in a given document by measuring the frequency of the unigram or bigram in a given document (i.e., text narrative) and the frequency of the unigram or bigram across all other text narratives. Finally, sparse terms, tokens appearing in only 0.05% of the documents, were removed to reduce noise and improve model efficiency.

#### Initial Training of the NLP Model

A random forest classifier consisting of 1,500 decision trees was trained on the first labeled dataset (*n* = 200, described above). Five-fold cross validation was performed to prevent overfitting to the data during model selection. K-fold cross validation (in our analysis, *k* = 5) is a model validation technique wherein the data is randomly separated into k bins; each bin is iteratively held out individually as a validation set while the remaining bins are used for model training (39). Model parameters were selected based on our prior work (7). The text classifier assigned each case in the unlabeled data a probability (range: 0–1) that it was DCAS. The performance of this model at identifying cases of DCAS in the unlabeled data was quantified using sensitivity (true positive rate), specificity (true negative rate), precision (positive predictive value), negative predictive value, and F1 score (harmonic mean of sensitivity and precision). The first iteration of the classifier using the manually labeled dataset (*n* = 200, 59 cases and 141 non-DCAS cases) had a sensitivity of 0.56, specificity of 0.90, precision of 0.70, and negative predictive value of 0.83, for an F1 Score of 0.62. The classifier correctly identified 56% of the true DCAS cases as DCAS; of all decedents identified as DCAS by the classifier, 70% of them were true DCAS cases. The classifier correctly identified 90% of the true non-DCAS cases as non-DCAS, and of all decedents identified as non-DCAS by the classifier, 83% of them were true non-DCAS cases. This iteration had excellent performance for non-DCAS, and mixed performance for DCAS cases.

#### Creation of the Second Labeled Data Set

A random subset of cases from the unlabeled data was selected across ranges of probabilities generated by the initial NLP classifier: i.e. 20 narratives each from the ≥0.8, <0.8 and ≥0.6, <0.4, and ≥0.2, <0.2 and ≥0 bins, and 30 narratives from the <0.6 and ≥0.4 bin to improve distinguishing borderline cases. This classifier-guided annotation process enables us to potentially identify additional examples not matching our initial keywords and to improve the model's ability to discriminate between examples. A total of 110 narratives were manually reviewed by TK via the same procedure and criteria as the first labeled dataset. Thirty-five DCAS were identified out of the 110 narratives; 14 DCAS were from the ≥0.8 bin, 11 from the <0.8 and ≥0.6 bin, and 10 from the <0.6 and ≥0.4 bin. These 110 labeled case narratives were then combined with the first labeled dataset, to create a final labeled data set (*n* = 310, 94 cases and 216 non-DCAS cases).

#### Second Iteration of the NLP Model

A random forest classifier consisting of 1,500 decision trees was trained on the second labeled dataset (*n* = 310). Again, 5-fold cross validation was performed to prevent overfitting to the data. The mean decrease in Gini coefficient, representing the importance of a given token for classification, was calculated for all tokens (40). Additional model performance metrics including sensitivity, specificity, precision, negative predictive value, and F1 were calculated. The second iteration of the classifier using the expanded, labeled dataset (*n* = 310, 94 cases and 216 non-DCAS cases) had a sensitivity of 0.57, specificity of 0.87, precision of 0.65, negative predictive value of 0.84, and a F1 score of 0.61. The classifier correctly identified 57% of the true DCAS cases as DCAS; of all decedents identified as DCAS by the classifier, 65% of them were true DCAS cases. The classifier correctly identified 87% of the true non-DCAS cases as non-DCAS, and of all decedents identified as non-DCAS by the classifier, 84% of them were true non-DCAS cases. This iteration had excellent performance for non-DCAS cases, and mixed performance for DCAS cases. This classifier was applied to the remaining unlabeled dataset.

#### Manual Review of Final Case Status

Decedent narratives identified as having a probability of ≥0.5 of being associated with driving cessation were manually reviewed by TK, in order to verify the predictions made by the classifier. Next, out of commitment to obtain the best approximation of the frequency of DCAS, we conducted a supplemental key-phrase search among the narratives identified by the classifier to have a <0.5 probability of being associated with driving cessation. This key-phrase search was informed by the classifier output and classifier training process. For a full list of keyword search criteria, see Supplementary Materials. Notably, these search terms were more specific and longer than those used in development of the first labeled dataset.

#### Qualitative Content Analysis of DCAS Narrative Texts

Finally, we used qualitative content analysis techniques to identify and characterize major themes from the narrative texts of DCAS cases (41). First, all DCAS narratives were collated and reviewed by TK, who took notes on the various features described in the texts. All texts were reviewed twice and notes were compared to ensure consistency. Salient features identified from this process were discussed with other members of the research team (VK and BM) to identify the central elements of each of the four themes: (1) health/functional decline (i.e., explicit statements about suicidal ideation or threats as a result of no longer being physically able to drive an automobile), (2) motor vehicle accidents (i.e., explicit mention of involvement in a car accident), (3) substance use (i.e., legal or disciplinary suspension or loss of license as a result of alcohol or substance use, family member intervention due to concerns about intoxicated driving), and (4) work/financial stress (i.e., anticipated or actual job loss as a result of being unable to drive, including those whose work entails commercial trucking and similar occupations). Next, all DCAS narratives were manually annotated to indicate which theme(s) they represented, with consultation with the broader research team as needed to resolve borderline cases. These themes were not mutually exclusive, and many DCAS cases referenced multiple or linked themes [i.e., legal suspension of license due to DUI (substance use) which resulted in job loss (work/financial stress)].

#### Statistical Analysis

Descriptive statistics were used to compare the demographic features of DCAS vs. non-DCAS as a function of sex, age, race, marital status, years of education, physical and mental health status, recent crisis, and the urbanicity of the county in which they resided. As age was expected to be associated with physical health status and marital status, we also conducted Breslow-Day Tests to assess homogeneity in their associations with DCAS across age groups. A Poisson regression was conducted to assess trends over time in the frequency of DCAS. This regression was limited to the 16 states that either began reporting or were already reporting data to the NVDRS in 2005, in order to minimize bias and data artifacts that may be present during a given state's first years of data reporting (7). These states included Alaska, Colorado, Georgia, Kentucky, Maryland, Massachusetts, New Jersey, New Mexico, North Carolina, Oklahoma, Oregon, Rhode Island, South Carolina, Utah, Virginia, and Wisconsin.

## Results

### Descriptive Findings

Within the NVDRS registry, from 2003 to 2017, there were 305 (0.04% of the analytic sample) suicides (*n* = 295) or undetermined deaths (*n* = 10) associated with driving cessation among adults 55 years and older (Table 1). The majority of cases of DCAS were male (84.9%) and non-Hispanic white (95.7%). Compared with deaths not related to driving cessation, DCAS cases were older (Mean Age 71.2 vs 66.6, *p* < 0.001), had fewer years of education (Mean 12.2 years vs. 12.9 years, *p* = 0.001), were more likely to have had physical health problems (55.4 vs. 38.8%, *p* < 0.001), were more likely to have had a recent crisis (45.6 vs. 21.1%, *p* < 0.001), and resided in more rural areas (Mean RUCC Score 3.04 vs. 2.94, *p* < 0.001). The associations between having physical health problems and DCAS, and between marital status and DCAS, were consistent between decedents younger than 65 and decedents 65 or older. For further descriptive details, see Table 1. The Poisson regression results suggest that among the states with at least 13 years of valid NVDRS data, the annual frequency of DCASs has not changed over time (Beta = 0.723, *p* = 0.407).

**Table 1:** Demographic characteristics of decedents of driving cessation associated suicide vs. decedents of non-driving cessation associated suicide.

	**Non-driving cessation associated suicide**	**Driving cessation associated suicide**	**Overall**	***p*-value**
	**(*N* = 65,637)**	**(*N* = 305)**	**(*N* = 65,942)**	
**Sex^a^**				0.002
Female	15,014 (22.9%)	46 (15.1%)	15,060 (22.8%)	
Male	50,623 (77.1%)	259 (84.9%)	50,882 (77.2%)	
**Age^b^**				<0.0001
Mean (SD)	66.6 (9.86)	71.2 (11.6)	66.6 (9.87)	
**Race^c^**				0.24
White	60,349 (91.9%)	292 (95.7%)	60,641 (92.0%)	
Black or African American	3,219 (4.9%)	8 (2.6%)	3,227 (4.9%)	
Asian/Pacific Islander	896 (1.4%)	2 (0.7%)	898 (1.4%)	
American Indian/Alaska native	299 (0.5%)	1 (0.3%)	300 (0.5%)	
Other/unknown	874 (1.3%)	2 (0.7%)	876 (1.3%)	
**Years of education^d^**				0.001
Mean (SD)	12.9 (2.90)	12.2 (2.71)	12.9 (2.90)	
**Marital status^c^**				0.001
Married/civil union/domestic partnership	28,046 (42.7%)	110 (36.1%)	28,156 (42.7%)	
Divorced	18,933 (28.8%)	86 (28.2%)	19,019 (28.8%)	
Widowed	9,874 (15.0%)	74 (24.3%)	9,948 (15.1%)	
Single or never married	7,781 (11.9%)	30 (9.8%)	7,811 (11.8%)	
Unknown or missing	1,003 (1.6%)	5 (1.6%)	974 (1.5%)	
**Physical health problem^a^**				<0.0001
No, not available, unknown	40,166 (61.2%)	136 (44.6%)	40,302 (61.1%)	
Yes	25,471 (38.8%)	169 (55.4%)	25,640 (38.9%)	
**Mental health problem^a^**				0.736
No, not available, unknown	38,394 (58.5%)	175 (57.4%)	38,569 (58.5%)	
Yes	27,243 (41.5%)	130 (42.6%)	27,373 (41.5%)	
**Recent crisis^a^**				<0.0001
No, not available, unknown	51,775 (78.9%)	166 (54.4%)	51,941 (78.8%)	
Yes	13,862 (21.1%)	139 (45.6%)	14,001 (21.2%)	
**RUCC rurality code^d^**				<0.0001
Mean (SD)	2.94 (1.52)	3.04 (1.44)	2.94 (1.52)	

a *Chi-Square Tests for Independence*.

b *Mann-Whitney's U-Test*.

c *Fisher's Test*.

d *Two-Sample T-Test*.

### Narrative Circumstances

Turning to the content of the text narratives of the DCAS narrative cases, there were several common features related to driving cessation. Texts describing a variety of external sources of pressure for driving cessation were noted, including a physician order, family members taking away keys/car/license, legal suspension or revocation of a license, and the Motor Vehicle Administrations (i.e., such as with a failure to pass an exam for license renewal). Others talked about abstaining voluntarily, due to feeling unsafe driving. Beyond discussions of voluntary vs. involuntary cessation, the salience of driving cessation among the DCAS cases varied substantially, and can be broadly categorized as *health/functional decline, motor vehicle accidents, substance use*, and *work/financial stressors*. We note that these categorizations are not mutually exclusive and that most DCAS narratives described multiple salient features, as well as factors unrelated to driving cessation. For descriptive details on DCAS cases, see Table 2.

**Table 2:** Description of driving cessation associated suicide decedents, by four themes identified via qualitative review.

	**Functional impairment**	**Accident**	**Substance use**	**Employment**
	(***n*** **= 157)**	(***n*** **= 43)**	(***n*** **= 68)**	(***n*** **= 26)**
**Sex**				
Female	18 (11.5%)	6 (14.0%)	10 (14.7%)	0 (0%)
Male	139 (88.5%)	37 (86.0%)	58 (85.3%)	26 (100%)
**Age**				
Mean (SD)	75.5 (10.8)	72.6 (11.4)	63.2 (7.64)	60.3 (3.92)
**Race**				
White	149 (94.9%)	40 (93.0%)	66 (97.1%)	24 (92.3%)
Black or African American	6 (3.8%)	2 (4.7%)	1 (1.5%)	2 (7.7%)
Asian/Pacific Islander	1 (0.6%)	1 (2.3%)	0 (0%)	0 (0%)
American Indian/Alaska native	0 (0%)	0 (0%)	1 (1.5%)	0 (0%)
Other/unknown	1 (0.6%)	0 (0%)	0 (0%)	0 (0%)
**Years of education**				
Mean (SD)	12.2 (2.83)	12.0 (3.27)	12.5 (2.19)	12.2 (0.447)
**Marital status**				
Married/civil union/domestic partnership	69 (43.9%)	14 (32.6%)	23 (33.8%)	12 (46.2%)
Divorced	32 (20.4%)	14 (32.6%)	24 (35.3%)	9 (34.6%)
Widowed	42 (26.8%)	12 (27.9%)	8 (11.8%)	0 (0%)
Single or never married	12 (7.6%)	2 (4.7%)	12 (17.7%)	4 (15.4%)
Unknown or missing	2 (1.3%)	1 (2.3%)	1 (1.5%)	1 (3.8%)
**Physical health problem**				
No, not available, unknown	29 (18.5%)	20 (46.5%)	54 (79.4%)	14 (53.8%)
Yes	128 (81.5%)	23 (53.5%)	14 (20.6%)	12 (46.2%)
**Mental health problem**				
No, not available, unknown	90 (57.3%)	24 (55.8%)	43 (63.2%)	16 (61.5%)
Yes	67 (42.7%)	19 (44.2%)	25 (36.8%)	10 (38.5%)
**Recent crisis**				
No, not available, unknown	90 (57.3%)	24 (55.8%)	28 (41.2%)	8 (30.8%)
Yes	67 (42.7%)	19 (44.2%)	40 (58.8%)	18 (69.2%)
**Disclosed suicidal ideation**				
No, not available, unknown	120 (76.4%)	33 (76.7%)	51 (75.0%)	15 (57.7%)
Yes	37 (23.6%)	10 (23.3%)	17 (25.0%)	11 (42.3%)
**History of suicidal thoughts**				
No, not available, unknown	119 (75.8%)	32 (74.4%)	54 (79.4%)	18 (69.2%)
Yes	38 (24.2%)	11 (25.6%)	14 (20.6%)	8 (30.8%)
**RUCC rurality code**				
Mean (SD)	2.99 (1.48)	3.35 (1.54)	2.93 (1.35)	3.15 (1.69)

*These four themes are not mutually exclusive, thus do not add up to N = 305*.

#### Health/Functional Decline and Motor Vehicle Accidents

In 13 (4.3%) of the DCAS cases, the narrative indicated that the decedent began reporting explicit suicidal ideation as a direct consequence of driving cessation (e.g., stating that they would kill themselves if they lost the ability to drive, would rather be dead than be unable to drive, or that there was no point to living without a license). In others, the texts described a general despondence about the consequences of deteriorating physical health, including driving cessation among other losses of independence (e.g., feeling depressed about declining health and inability to drive). For example, 157 of the DCAS texts explicitly indicated functional impairments, both in physical and mental health (i.e., cardiovascular events, vision loss, chronic pain, Parkinson's disease, Alzheimer's disease, dementia, seizures, depression, and anxiety), as precipitating driving cessation. Forty-three DCAS cases had been in a car accident prior to their suicide; twenty of these had explicit indication of functional impairments contributing to driving cessation and/or the accident. Both of these experiences were more common among older decedents: the average age of DCAS decedents who had functional impairment was 75.5 as compared to 66.8 of other DCAS (*p* < 0.001); the average age of DCAS decedents who had an accident was 72.6 as compared to 71.0 of DCAS decedents who did not have an accident (*p* < 0.001).

#### Substance Use

Substance use is a known correlate of suicidal behavior, and it also played a role in driving cessation. Sixty-eight (22.3%) of the DCAS narratives included explicit description of alcohol related driving cessation. These included longer term cessation such as DUI license suspensions and revocations, as well as shorter term pressures such as family members withholding keys and vehicle access from the intoxicated decedent. The average age of alcohol-related DCAS cases was significantly younger than that of non-alcohol related DCAS decedents (Mean age 63.2 vs 73.5, *p* < 0.001).

#### Work/Financial Stressors

Finally, occupational stressors as a consequence of driving cessation were also noted; for example, there were 26 DCAS cases in which the decedent lost their job or feared job loss as a result of driving cessation (Table 2). The majority of these decedents were truck drivers who had lost their commercial driver's license due to physical health problems or DUIs. Other occupational circumstances involved a lack of reliable transport for a job leading to loss of employment. The average age of decedents whose driving cessation affected employment was significantly lower than that of decedents whose drive cessation did not affect their employment (Mean age of 60.3 vs. 72.3, *p* < 0.001).

### Methodological Findings

When applied to the full unlabeled dataset, the second iteration of the classifier identified 382 narratives as having a ≥0.50 probability of being associated with driving cessation among the remaining 65,632 unlabeled decent narratives. Of these 382, a manual review identified 116 (30.37%) as true-positives, with the remaining 266 decedents as false-positives. The non-systematic, supplementary key-phrase search through the remaining 65,250 narratives indicated as having a <0.50 probability of DCAS, yielded 95 additional DCASs as false negatives. When assuming that the supplemental key-phrase search captured all false negatives, albeit a slight underestimate, the final iteration of the model had a sensitivity of 0.55, specificity of 0.99, precision of 0.30, a negative predictive value of 0.99, and an F1 score of 0.39. The final classifier correctly identified 55% of the true DCAS cases as DCAS; of all decedents identified as DCAS by the classifier, 30% of them were true DCAS cases. The final classifier correctly identified 99% of the true non-DCAS cases as non-DCAS, and of all decedents identified as non-DCAS by the classifier, 99% of them were true non-DCAS cases. This final iteration had excellent performance for non-DCAS, and mixed performance for true-DCAS. The ten tokens with the greatest mean decrease in Gini coefficient, i.e., importance for classification, were “licens[e],” “drive,” “longer,” “state,” “check,” “area,” “vision,” “abil[ity],” “found deceas[ed],” and “use.”

## Discussion

This study aimed to quantify suicide cases related to driving cessation in the NVDRS, a previously unmeasured phenomena in this registry, by applying NLP, a data science tool appropriate for classifying large amounts of textual data. The primary findings of this study are that (1) although rare, suicide related to driving cessation does occur among middle-age and aging adults; (2) cases of suicide associated with driving cessation were older, more likely to be male, be widowed, have physical health problems, have had a recent crisis, and resided in more rural counties compared to suicide deaths not related with driving cessation; and (3) given the diverse range of terms and phrases used to indicate driving cessation in the narratives, there are additional challenges to developing an random forest text classification model to identify DCAS in these data.

A greater proportion of DCAS decedents were widowed than non-DCAS decedents. Ang et al. (42) found that older couples develop strategies, such as sharing driving duties, splitting between the more “active” role as the driver and the more “passive” role as the passenger, and regulating each other's behavior to ensure safe driving. In addition to the bereavement, being widowed could conceivably increase suicide risk via the large disruption to an aging adult's social network and ability to participate in daily routines (43). Further, similarly to driving cessation, history of an ongoing physical health problem related to ability to operate a vehicle was also associated with DCAS (13, 44–46). It is conceivable that both of these associations were due to the age of decedents of DCAS, such that there is greater likelihood of a deceased spouse and accumulation of physical health problems with increasing age, although this analysis showed these findings were consistent for decedents <65 years old and those who were 65 or older.

This analysis illustrated the salience of being in a car accident as a preceding factor, rather than as a means of self-harm, for suicide. For example, there were cases in which a decedent was involved in a car accident and were told they may have their license revoked on the morning of their suicide. Other cases described a similar scenario, but with a much longer gap in time between the accident and suicide, such as several weeks to months. Other examples include adult offspring trying to prevent their parents from driving after they had gotten into a motor vehicle accident. The psychological distress and depression commonly following motor vehicle accidents (47), as well as the acute “reminder” of aging (especially when the accident is due to functional impairment) and potential loss of social role as a “driver” may lead to the intense affective states that precede a suicide crisis (21, 47, 48).

Alcohol related driving cessation was identified in just under a quarter of the DCAS decedents, more commonly among younger DCAS decedents. Alcohol abuse is independently associated with suicide risk and depression (49). Of note, approximately half of the alcohol-related DCAS decedents had experienced or anticipated employment consequences, primarily through loss of a Commercial Driver's License. Thus, decedents of DCAS may have experienced increased distress not only directly due to the effects of alcohol abuse and mental health sequelae of driving cessation, but also due to loss of (or anticipated loss of) employment. Previous studies have found that work stress, limited physical activity and healthcare access, and sleep deprivation place long haul truck drivers at risk for substance abuse (50). Improved work conditions to reduce stress and sleep deprivation, telehealth appointment access, and concerted efforts for substance abuse counseling may reduce risk of alcohol abuse-related driving cessation, potentially leading to greater road safety, mental health, and job security among truck drivers (51).

Driving is the primary means of transportation in the US, particularly in more rural areas. The counties where decedents of DCAS lived were on average marginally more rural than those of decedents of non-DCAS. Older drivers residing in rural areas rate the ability to drive to be more important, and the loss of the ability to drive to be more impactful, than older drivers from urban areas (52). Transportation access is lower in rural areas, and reliance on informal social support networks becomes particularly important among aging adults who have voluntarily ceased driving (53, 54). Although our analysis is not causal and further research is necessary to substantiate this notion, it is conceivable that DCAS is more common in rural areas where driving cessation is considered to be more impactful, and the deleterious mental health effects may be magnified.

Findings call attention to the similarities, and differences, in correlates of driving cessation as compared to the correlates of suicide. Even among suicide deaths, those that are associated with driving cessation are more likely to be male and have health problems. Although not directly testable with our data, this finding may suggest that functional impairment (i.e., no longer being able to drive) helps explain why health problems are associated with suicide (55). While driving cessation is more common among women than men (56–59), studies suggest that driving cessation fractures social connections for men more than for women (31, 60). Further, aging adults belonging to minority racial groups have also been found to be more likely to stop driving, although their risk of suicide is much lower than that of non-Hispanic whites (56, 57). Overall, the present analysis suggests that correlates of DCAS may be more similar to risk factors for suicide, rather than correlates of driving cessation.

### Methodological Considerations

The dataset used in this research contained over 65,000 text case narratives. Qualitative data of this scale requires additional analytic tools. In this study, we employed a supervised NLP algorithm to efficiently identify cases related to driving cessation. Although this methodology was efficient, the NLP classifier had mixed performance. The specificity and negative predictive value were excellent, whereas sensitivity and precision were poor, indicating the model likely underestimates the total number of driving-cessation related cases in the data. We believe this performance was jointly due to the scarcity of the outcome and the heterogeneity in written descriptions of driving cessation, which provide limited textual features for recognizing true positive examples of driving cessation. NLP algorithms can have substantial strengths in classification of text data such as high accuracy due to ensemble learning and rapid handling of very large, multi-dimensional datasets (61). However, severe class imbalance, that is, few cases relative to non-cases (i.e., in our situation, the <1% prevalence of DCAS), is a challenge for NLP algorithms (62). We attempted to mitigate this issue by training the model on an initially labeled dataset that had an approximate 1:3 split of DCAS cases to non-DCAS cases.

The algorithm's mixed performance may be partially attributable to the model's over-reliance on the keywords used to develop the initial labeled dataset. This hypothesis is corroborated by three of the four keywords “license,” “drive,” and “vision” having very high importance in the model classification process, per their high mean decreases in the Gini coefficient. Further, written descriptions of driving cessation were quite variable; it is possible that the use of bigrams was insufficient in capturing driving cessation (i.e., the lexicon of driving cessation is not unique). To address this issue, we conducted a supplemental key-phrase search after applying the NLP classifier, which did identify a substantial portion of additional DCAS cases. Assuming that the supplemental key-phrase search identified the remaining DCAS in the NVDRS, albeit an underestimate, the sensitivity of the final classifier exceeded the precision. This suggests that the proportion of true DCAS that the classifier failed to identify is less than the proportion of cases incorrectly identified as DCAS. Indeed, most DCAS narratives including those identified by the NLP classifier and those only found via the supplemental key-phrase search, contained at least one of the aforementioned keywords, and fewer of these narratives were written without the keywords in manners such as “did not have transportation,” “loss of driving privileges,” or “lost his car and could not get it back” (moderate sensitivity). However, narratives in the NVDRS that were irrelevant to DCAS, yet contained these keywords, were more common, as evidenced by the keyword search conducted to develop the initial labeled dataset (low precision). In sum, this analysis shows that while NLP tools can be efficient methods for handling large amounts of qualitative data, the accuracy in this study was hindered by the extreme rarity of the outcome and variable descriptions of driving cessation in the narratives. Alternatives to NLP approaches have different strengths and limitations, including efficiency identifying rare and emergent cases, as well as interpretability (63–65).

### Strengths and Limitations

To our knowledge, the frequency of DCAS among aging adults has never been estimated. The combined methodology of the NLP classifier and supplemental key-phrase search enabled us to quantify this phenomenon, characterize cases of suicide associated with driving cessation, and highlight the various precipitating factors that relate to driving from a large mortality registry. Findings should be interpreted in light of study limitations. Although NLP is an efficient method for analyzing large amounts of text, in this study the classifier had limited accuracy due to the rarity of the outcome and heterogeneity in descriptions of driving cessation. The large sample size and number of hypothesis tests may have led to the discovery of spuriously significant associations. Although economic and social resources are likely protective against suicide risk among individuals who have ceased driving, we were not able to incorporate these variables due to lack of data. Additionally, the NVDRS data consolidated “No,” “Not Available,” and “Unknown” for some of the variables, which may be biased to report no results. Lastly, although standardized training and criteria exist, the medical examiner text narratives are limited to information deemed relevant by the NVDRS coders. Thus, it is possible that there are suicide decedents for whom driving cessation was a factor in their suicide, but this information was not gathered or not indicated by the coders, leading to an underestimate in DCAS.

## Conclusion

In this registry of suicide deaths, older age, marital status, physical health problems, functional impairment, alcohol-related driving cessation, history of a car accident, and potential loss of employment were noted among cases associated with driving cessation. This study adds to the body of research describing the importance of mobility for physical and mental health, quality of life, and social connectedness among aging adults. Efforts to promote mobility in later life include improving alternative transportation access for aging adults, especially in rural areas, as well as encouraging preemptive planning for driving cessation—such as the CDC's evidence-based “MyMobility Plan” —may reduce the impact of driving cessation on aging adults and their families (66–69).

Importantly, this analysis does not mitigate or suggest eliminating license renewal regulations for aging adults as a component of public transportation safety. Instead, findings call attention to the fact that individuals and agencies with the power to revoke an aging adult's ability to drive, including physicians, licensing bodies, or family members, should be mindful of the implications of driving cessation on overall quality of life. As noted by Aronson (20), “Matters such as driving retirement that have both individual and societal consequences are no less worthy of clinical attention than diseases…they should be accorded training, time, and remuneration commensurate with their complexity.”

## Data Availability Statement

Publicly available datasets were analyzed in this study. This data can be found at: https://www.cdc.gov/violenceprevention/datasources/nvdrs/index.html. Restricted Access Narrative Data require a proposal.

## Ethics Statement

The studies involving human participants were reviewed and approved by University of Michigan Institutional Review Board. Written informed consent for participation was not required for this study in accordance with the national legislation and the institutional requirements.

## Author Contributions

TK developed the concept for the manuscript, planned analyses, conducted analyses, and drafted the manuscript. VK and DJ planned analyses and assisted with drafting the manuscript. BM developed the concept for the manuscript and drafted the manuscript. All authors contributed to the article and approved the submitted version.

## Conflict of Interest

The authors declare that the research was conducted in the absence of any commercial or financial relationships that could be construed as a potential conflict of interest.

## Publisher's Note

All claims expressed in this article are solely those of the authors and do not necessarily represent those of their affiliated organizations, or those of the publisher, the editors and the reviewers. Any product that may be evaluated in this article, or claim that may be made by its manufacturer, is not guaranteed or endorsed by the publisher.
