# Supplementary material for: A Data Science Approach to Estimating the Frequency of Driving Cessation Associated Suicide in the US: Evidence From the National Violent Death Reporting System
Source: Front Public Health. 2021 Aug 16;9:689967. doi: 10.3389/fpubh.2021.689967 (PMC8415628; doi:10.3389/fpubh.2021.689967)
Supplement: Supplementary file 1 [file Table_1.DOCX]

**Supplemental Key-Phrase Search**

In light of the low sensitivity of the random forest classifier, we searched remaining non-DCAS cases for additional driving cessation associated suicides. The following key-phrase searches were used.

give up keys

give up vehicle

give up car

away keys

privil

away his keys

away her keys

unable to drive

quit driving

stopped driving

stop driving

couldn't drive

dmv

couldn't drive

cannot drive

Department of Motor,

onger drive

ability to drive

allowed to drive

for transportation

revoke

impounded

lost his car

hidden keys

taken his car

taken her car

transportation

not drive

able to drive

vehicle administration

a driver

license

privel

doesn't drive

want to drive

wanted to drive

homebound,

could drive

would drive

up keys

away key

give up

up his car

up his keys

up her car

up her keys

taken his keys

taken his car

taken
